# Probiotic therapy - recruiting old friends to fight new foes

**DOI:** 10.1186/1757-4749-2-5

**Published:** 2010-06-25

**Authors:** Roy D Sleator

**Affiliations:** 1 Department of Biological Sciences, Cork Institute of Technology, Rossa Avenue, Bishopstown, Cork, Ireland; 2 Alimentary Pharmabiotic Centre, University College Cork, Ireland

## Abstract

Against a backdrop of increasing antibiotic resistance, and the emergence of new and evolving pathogens, clinicians are increasingly forced to consider alternative therapies - probiotics are one such alternative.

## The problem

With life-cycles measured in minutes as opposed to years, bacteria have an extraordinary ability to evolve and adapt rapidly to changes in their environment [1]. Thus, in a world where only the fittest survive, those bacteria which have developed resistance to antibiotics will predominate. This is particularly apparent in hospital environments where bacteria are in constant contact with many different antibiotics; such repeated exposure has facilitated the development of multiple antibiotic resistance and the emergence of ever more virulent nosocomial infections.

## Probiotic Therapy - a possible solution?

**Figure 1:**
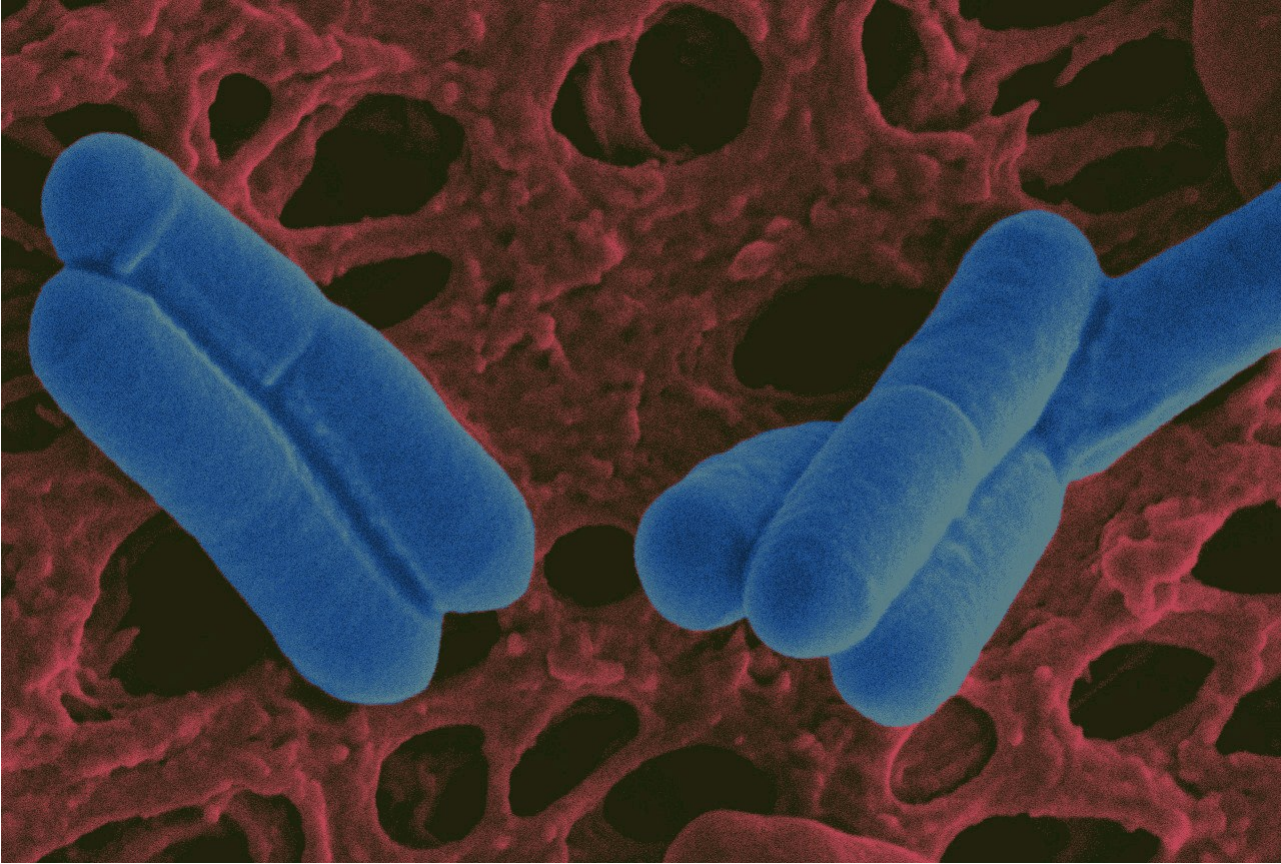
**Scanning electron micrograph of the probiotic strain *Lactobacillus salivarius *UCC118 at a magnification of 25,000 ×.** False colour added by Pat Casey.

**Figure 2:**
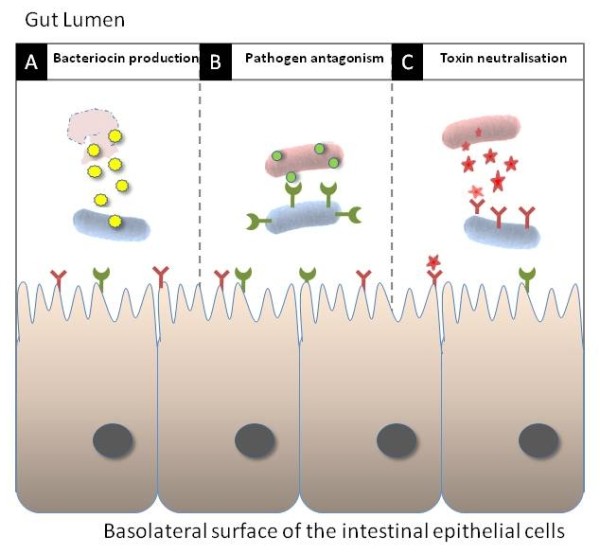
**Overview of the anti-bacterial potential of designer probiotics**. Bacteriocin produced by the probiotic (blue) can lyse invading pathogens (red) (A) while heterologously expressed receptor mimics on the surface of probiotic cells can antagonise pathogen adherence to the host (B) and neutralise toxin production (C). Imaged reproduced, with permission, from [18].

Faced with an emerging pandemic of antibiotic resistance, clinicians and scientists alike are now struggling to find viable therapeutic alternatives to our failing antibiotic wonder drugs [2]. One such alternative may be bacteria themselves - the application of probiotics; so called "good bugs" (Fig. 1), for therapeutic effect [3,4]. While the exact mechanisms by which probiotic bacteria inhibit pathogens are as yet poorly understood, some advances have nevertheless been made in our understanding of probiotic function [5]. In addition to competing with pathogens for niches and nutrients, "competitively excluding" disease causing microbes from the host [6], certain probiotic bacteria have also been shown to produce potent antimicrobial peptides (bacteriocins) which specifically target the invading pathogen [7] (Fig. 2A). While traditional antibiotics usually exert their activities *via *a specific mode of action; for example, penicillin interferes with the cross-linking of two linear polymers by inhibiting the transpeptidase reaction, bacteriocins on the other hand have quite diverse activities. Nisin and many other structurally related lantibiotics for example, use the cell wall precursor lipid II bound to the membrane as a docking molecule for pore formation and combine at least two modes of action, i.e., pore formation and inhibition of cell wall biosynthesis, for antibacterial activity at nanomolar concentrations [8]. These multiple modes of action significantly reduce (but do no eliminate) the risk of resistance development [9].

However, despite their potent anti-pathogenic effect, a significant limitation of this approach is that probiotic bacteria tend to be physiologically fragile; often not surviving to sufficiently high numbers during prolonged storage in delivery matrices such as foods (yogurt and probiotic drinks) or tablet formulations [10]. Furthermore, following ingestion, the already depleted probiotics must face the considerable physiological defences of the host (gastric acidity, bile, low iron, elevated osmolarity and temperature) in order to colonize the gastrointestinal tract in sufficient numbers to exert a therapeutic effect [11,12].

## Patho-biotechnology - making good bugs better

One approach to improving the physiological robustness and stress tolerance of probiotic strains is patho-biotechnology [13,14]. Essentially, this novel approach involves the generation of "improved" probiotic strains, using stress survival systems mined from more physiologically robust pathogenic microbes [15]. The physiological versatility of pathogenic genera, oscillating between the external environment and the host, makes them a veritable treasure trove of genes that could potentially be used to improve the technological robustness of less well adapted probiotic strains [16]. Indeed, recent work in our laboratory has shown that cloning and heterologous expression of a single bile resistance gene, from the food borne pathogen *Listeria monocytogenes *in the probiotic strain *Bifidobacterium breve*, not only improves gastrointestinal colonisation and persistence, but also significantly bolsters the clinical efficacy of the probiotic strain [17].

## Therapy

In addition to improving their physiological stress tolerance, resulting in improved delivery and persistence within the gut, recent studies have led to the development of 'designer probiotics' which specifically target enteric infections by blocking crucial ligand-receptor interactions between the pathogen and its target host cell [10,18,19]. Many disease causing bacteria exploit oligosaccharides displayed on the surface of host cells as receptors for toxins and/or adhesions, enabling colonization of the mucosa and entry of the pathogen or secreted toxins into the host cell. Blocking this adherence prevents infection (Fig. 2B), while toxin neutralization ameliorates symptoms until the pathogen is eventually overcome by the host immune system (Fig. 2C). 'Designer probiotics' have been engineered to express receptor-mimic structures on their surface [20]. When administered orally these probiotics bind to and neutralize toxins in the gut lumen and interfere with pathogen adherence to the intestinal epithelium - thus essentially "mopping up" the infection. A particularly attractive feature of this toxin neutralisation strategy is that, unlike antibiotic therapy, it applies no selective pressure for evolution of resistance by the pathogen. Blocking toxin mediated host injury by the receptor mimic would negatively affect the capacity of the pathogen to survive and reproduce. Furthermore, mutations in a toxin sequence that prevents binding to a receptor mimic would logically also prevent the toxin from interacting with its natural target, thereby attenuating virulence. Therefore, widespread use of such agents in a therapeutic setting should have negligible long-term adverse consequences. As well as treating enteric infections, 'designer probiotics' are among the most recent conscripts in the war against AIDS, expressing HIV receptors which compete with host cell receptors for the virus, thus providing a natural innate barrier to HIV attachment and infection [21].

## Prophylaxis

**Figure 3:**
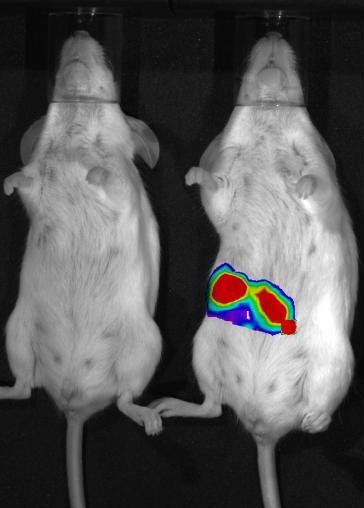
**Mice orally infected with a luminescent bacterial pathogen (*Listeria monocytogenes*)**. The mouse on the left was subsequently fed with a probiotic (*Lb. salivarius *UCC118), which inhibited the pathogen, thus quenching the light. The mouse on the right was instead given a placebo, which had no effect on infection or light production. The image was taken by Pat Casey using the IVIS Imaging System 100 Series from Xenogen.

In addition to infection control (Fig. 3), probiotics can also be engineered to function as novel vaccine delivery vehicles which can stimulate both innate and acquired immunity, but lack the possibility of toxicity which exists with more conventional vaccines that rely on live attenuated pathogens [22]. Probiotic vaccine carriers administered by the mucosal route (i.e. orally or by nasal spray) mimic the immune response elicited by natural infection and can lead to long lasting protection. Mucosal vaccine delivery also offers significant technological and commercial advantages over traditional formulations including: reduced pain and the possibility of cross contamination associated with intramuscular injection, as well as the lack of necessity for medically trained personnel to administer the vaccine - important considerations for large scale vaccination protocols in less well developed countries. Furthermore, not only do probiotics circumvent *in vivo *sensitivity to gastric acidity associated with oral application of therapeutic or prophylactic compounds, they can also be produced cheaply; grown to high levels, dried and stored for years at ambient temperatures.

## Beyond conventional antibiotic therapies

In conclusion then, "designer probiotics" can be engineered to kill pathogens, neutralise toxins, and facilitate re-colonisation of the resident beneficial microflora while at the same time priming both the innate and adaptive immune system; strengthening the host against subsequent infection - an approach far beyond the reach of conventional antibiotic therapies. Thus, the war against the antibiotic resistant "super bugs" may eventually be won by recruiting engineered "good bugs" as our allies.

## Author's information

Sleator is Editor-in-Chief of the peer reviewed scientific journal *Bioengineered Bugs *http://www.landesbioscience.com/journals/biobugs.
